# A case of corticosteroid-dependent recurrent pericarditis with different response to two IL-1 blocking agents

**DOI:** 10.1186/1546-0096-13-S1-P155

**Published:** 2015-09-28

**Authors:** K Theodoropoulou, A von Scheven-Gête, S Bressieux-Degueldre, M Prsa, F Angelini, T Boulos, M Hofer

**Affiliations:** 1 Department of Pediatrics, Lausanne University Hospital (CHUV), Lausanne, Switzerland

## Introduction

Recurrent pericarditis (RP) has a controversial pathogenesis that crosses infectious, auto-immune and auto-inflammatory pathways. It has been suggested that in some cases it might be an unrecognized auto-inflammatory disease. Recent studies have demonstrated that anakinra, an interleukin-1 receptor antagonist (IL-1RA), represents an effective treatment for the control of corticosteroid-dependent cases.

## Objectives

Here we describe a case of cortico-dependent recurrent pericarditis with a different response to two IL-1 blocking agents, anakinra and canakinumab.

## Materials and methods

Case report.

## Results

A 11-year-old boy was admitted to our hospital with acute precordial pain, orthopnea, fever and increased levels of acute phase reactants. Acute pericarditis was confirmed by echocardiography and a treatment with prednisone was started with prompt clinical improvement. Pericarditis recurred twice during steroid tapering (at 1mg/kg/day and 0.5mg/kg/day respectively). After exclusion of infectious origin, therapy with anakinra (2mg/kg/day) was established (to avoid long term steroid side effects) followed by dramatic clinical response and normalisation of laboratory findings despite tapering and discontinuation of prednisone. Treatment with anakinra was discontinued after 5 months with recurrence of pericarditis one week later. Anakinra was resumed with an excellent response. Five months later, while being in complete remission, anakinra was replaced with canakinumab (2mg/kg/dose) due to patient's intolerance of daily injections. One week later, the patient experienced a new episode of pericarditis requiring corticotherapy. Two more relapses occured during steroid tapering, after 6 weeks and 2 months, in spite of the uptitration of canakinumab to 4mg/kg/dose. Anakinra was restarted with prompt clinical and biological remission and prednisone was discontinuated without recurrence of pericarditis. Four weeks later, anakinra was spaced out every 2 days and a treatment of colchicine was added. After further 12 weeks follow-up under anakinra and colchicine, the pericarditis is still in remission.

## Conclusion

We describe a case of steroid-dependent RP with a dramatic therapeutic response to IL-1RA (anakinra) but without response to IL-1β monoclonal antibody (canakinumab). This unexpected observation could suggest that Il-1α might have a role in the pathogenesis of RP. A more precise usefulness of each IL-1 blocking agent requires confirmation in prospective controlled trials.

## Consent to publish

Written informated consent for publication of their clinical details was obtained from the patient/parent/guardian/relative of the patient.

